# The effects of abused drugs on ferroptosis pathways: potential therapeutic targets for substance use disorders

**DOI:** 10.3389/fncel.2026.1899553

**Published:** 2026-09-03

**Authors:** Tamara Bouchard, Brooke Russell, Liam Liyang Guo, Tina Nguyen, Yan Cheng, Yan Y. Sanders, Ming-Lei Guo

**Affiliations:** 1 Department of Biological and Translational Department, Macon and Joan Brock Eastern Virginia Medical School/Virginia Health Sciences, Old Dominion University, Norfolk, VA, United States; 2 Keck School of Medicine of the University of Southern California, Los Angeles, CA, United States; 3 Center for Integrative Neuroscience and Inflammatory Diseases, Eastern Virginia Medical School/Virginia Health Sciences, Old Dominion University, Norfolk, VA, United States

**Keywords:** ferroptosis, neurodegeneration, neuroinflammation, ROS, substance use disorder

## Abstract

Substance use disorders (**SUDs**) remain major public health concerns worldwide, particularly in developed countries. SUDs are characterized by persistent neuroinflammation and synaptic dysfunction in the brain. Despite decades of extensive investigation, the detailed mechanisms underlying SUDs remain elusive. Ferroptosis is a highly regulated cell death process deeply affected by iron metabolism, lipid peroxidation, reactive oxygen species (**ROS**) production, and antioxidant systems. It has been implicated in multiple neurodegenerative diseases, including Alzheimer's disease, Parkinson's disease, multiple sclerosis, and ischemic stroke. Recently, emerging evidence has highlighted the role of ferroptosis in drug-induced pathological changes. Various types of abused substances including alcohol, cocaine, methamphetamine (**METH)** nicotine, cannabis, and opioids have been shown to disrupt ferroptosis-relevant pathways, leading to microglial activation and neuronal injury. Inhibition of ferroptosis could mitigate these pathological changes in preclinical models. Here, we summarize current evidence demonstrating the effects of abused drugs on ferroptosis pathways. Collectively, these findings underscore the potential role of ferroptosis in the pathophysiology of SUDs. Targeting ferroptosis may represent a promising therapeutic strategy for alleviating drug-induced neurological damage and improving recovery outcomes such as cognitive and memory deficiency in individuals with addiction.

## 1 Introduction

SUDs represent a persistent global public health challenge, particularly in modern societies. Epidemiological studies estimate that more than 35 million individuals are affected by drug use disorders, frequently accompanied by neurological complications such as cognitive impairment, memory deficits, and behavioral abnormalities (Volkow and Blanco, 2023; Connery et al., 2020; Uhl et al., 2019). Among them, alcohol and opioid misuse are major contributors to morbidity and mortality (Sanchez-Roige et al., 2022). Beyond their health effects, SUDs exert profound societal impacts, including increased unemployment, crime, family instability, and homelessness, resulting in substantial economic burden (Daley, 2013). Despite decades of intensive basic and clinical research, the mechanisms underlying SUD pathogenesis remain incompletely understood. Notably, there are still no FDA-approved pharmacotherapies for certain addictions, such as cocaine use disorders (**CUDs**) (Schwartz et al., 2022). Therefore, identifying shared molecular pathways disrupted by different classes of abused drugs is essential for developing more effective therapeutic strategies.

Ferroptosis, a regulated form of cell death driven by iron-dependent lipid peroxidation, has recently emerged as a key mechanism linking iron dysregulation, oxidative stress, neuroinflammation, and neurodegeneration (Liu and Zeng, 2026; Christodoulou et al., 2026). Ferroptosis has been implicated in diverse pathological conditions, including viral infections, ischemic stroke, and neurodegenerative diseases (Wang L. et al., 2026; Zhang et al., 2026; Sun et al., 2026; Tao et al., 2026; Sandra Monserrat et al., 2026). Growing evidence indicates that various types of abused substances could interact with ferroptosis-related pathways, elevating ROS production, disrupting iron homeostasis, and impairing antioxidant defenses in the central nervous system (**CNS**) which contribute to microglial activation and neuronal injuries (Bo et al., 2023; Guo et al., 2022). Of note, pharmacological inhibition of ferroptosis alleviates neuroinflammatory responses and neuronal damages in multiple preclinical models (Khan et al., 2025). In this review, we summarize current evidence revealing how abused drugs modulate ferroptosis relevant pathways, with a focus on iron metabolism, ROS generation, lipid peroxidation, and antioxidant systems. Collectively, these findings suggest that ferroptosis may represent a convergent mechanism underlying drug-induced neurotoxicity and serves as a promising target for therapeutic intervention in SUDs.

## 2 Overview of ferroptosis

### 2.1 Ferroptosis pathways

**Figure 1:**
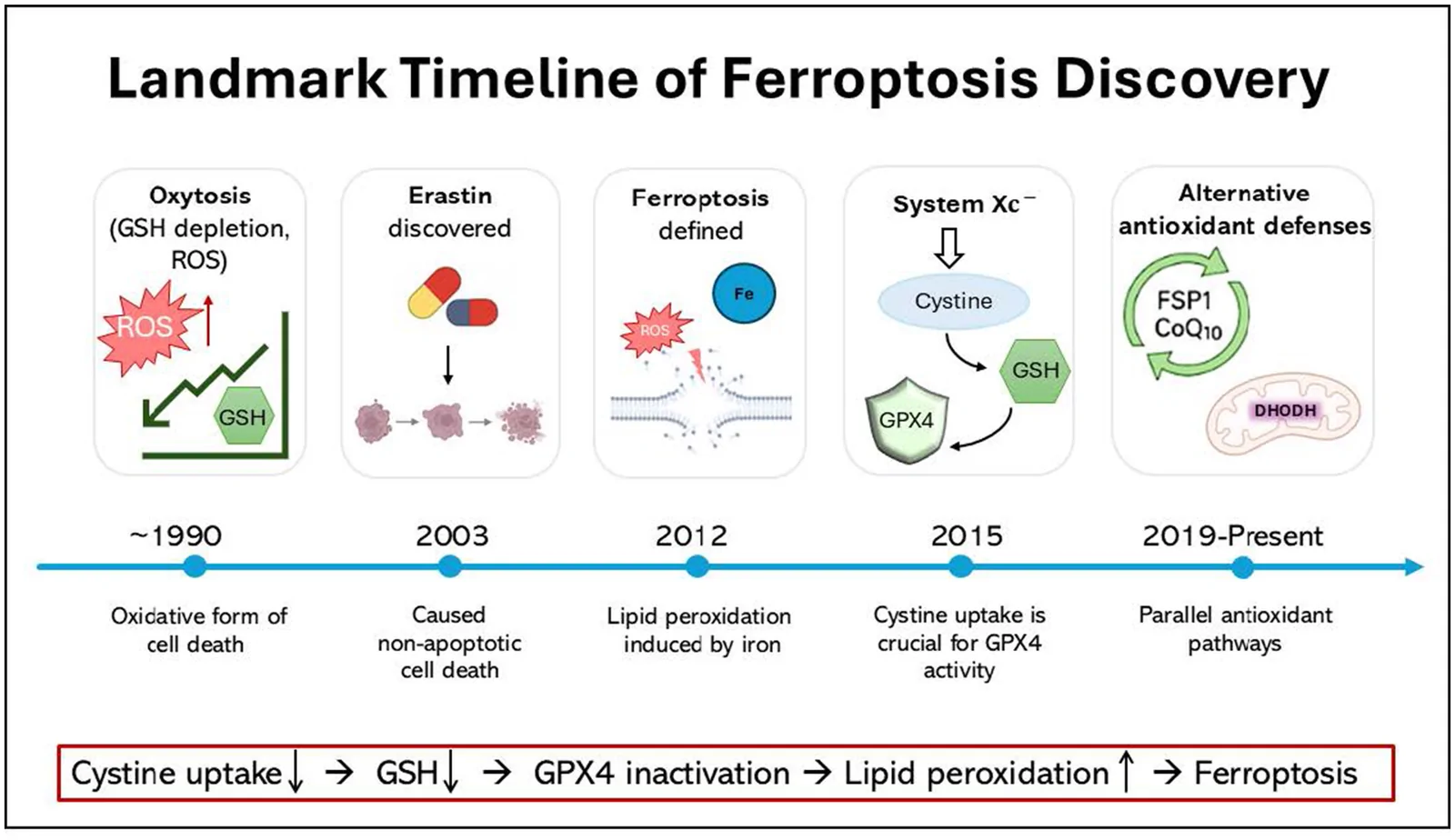
Key milestones in the discovery of ferroptosis. 1989 - 2001: Early “oxytosis” observations: Studies of glutamate-induced cell death in neuronal cells revealed a non-apoptotic, oxidative form of death characterized by GSH depletion and lipid peroxide accumulation; **2003:** Identification of erastin: A small molecule (erastin) was discovered to selectively kill RAS-mutant cancer cells through a non-apoptotic mechanism, initiating systematic investigation into this atypical cell death pathway (Dolma et al., 2003). **2008 - 2011:** Discovery of ferroptosis-inducing compounds: RSL3 was identified, and researchers noted hallmarks including iron dependence and lipid peroxide accumulation, distinguishing this process from known cell death types. **2012:** Formal definition of ferroptosis: Dixon coined the term ferroptosis defining it as an iron-dependent form of regulated cell death driven by lipid peroxidation (Yang et al., 2014). **2014:** GPX4 identified as a central regulator for ferroptosis and that its inhibition directly triggers lipid peroxide accumulation and cell death (Gaschler et al., 2018). **2014 - 2015:** System Xc^−^- GSH axis established: The cystine/glutamate antiporter (system Xc^−^) and GSH metabolism were linked to ferroptosis control, explaining how erastin induces ferroptosis by depleting intracellular cysteine and GSH. **2016 - 2017:** Lipid metabolism determinants acyl-CoA synthetase long-chain family member 4 (ACSL4): ACSL4 and polyunsaturated phospholipids determine ferroptosis sensitivity, establishing lipid composition as a critical factor in ferroptotic susceptibility. **2017:** Integration into broader biology: A comprehensive review positioned ferroptosis as a central nexus linking iron metabolism, redox biology, and lipid metabolism, highlighting its roles in disease and physiology (Stockwell et al., 2017). **2019 - present:** Expansion of regulatory networks: Novel pathways, including FSP1-CoQ10, DHODH, and GCH1-BH4 systems, were identified as parallel antioxidant defenses, broadening the mechanistic framework and therapeutic relevance of ferroptosis (Wang J. et al., 2026).

Ferroptosis is a type of cell death process coordinately regulated by multiple signaling pathways. Since its formal description in 2012, ferroptosis has been increasingly acknowledged to play critical roles in various diseases including cancers, virus infection, stroke, and multiple neurodegenerative diseases (Wang L. et al., 2026; Zhang et al., 2026; Sun et al., 2026; Tao et al., 2026; Sandra Monserrat et al., 2026). Since there have already been excellent review papers discussing the complex mechanisms underlying ferroptosis regulation, we here just briefly outline the landmark events for ferroptosis discovery during the last three decades (Figure 1) and summarize the most well-known pathways critical for ferroptosis and deeply affected by abused drugs.

**(1) Iron homeostasis**: The cellular iron concentration is tightly regulated to keep balance due to its essential roles in metabolism with its potential toxicity (Muckenthaler et al., 2017). Iron is primarily imported into cells via transferrin receptor (**TfR**)-mediated endocytosis as Fe^3+^ status, which is subsequently reduced to Fe^2+^ and released into the cytosolic labile iron pool. Intracellular iron levels are mainly controlled through storage in ferritin in a non-reactive form, and export via ferroportin. Iron regulatory proteins (**IRP1/2**) are the key regulators of cellular iron balance, modulating the expression of TfR, ferritin, and ferroportin. Disruption of this regulatory network leads to intracellular iron accumulation, which can catalyze Fenton reactions to generate ROS and promote oxidative damage, thereby contributing to ferroptosis process.

**(2) ROS production**: ROS are highly reactive, oxygen-containing chemical molecules mainly including superoxide anion (${\text{O}}_{2}^{•-}$), hydrogen peroxide (H_2_O_2_), hydroxyl radical (^•^OH), singlet oxygen, lipid hydroperoxides, and peroxynitrite (ONOO^−^). Several cellular origins of ROS have been identified. One major source is mitochondrial electron transport chain (**ETC**), where electron leakage during oxidative phosphorylation leads to ${\text{O}}_{2}^{•-}$ formation (Shadel and Horvath, 2015). This is accompanied by the change in ultrastructural features of mitochondria which in ferroptosis exhibits smaller (shrunken) morphology with increased mitochondrial membrane density and reduced or absent cristae (Stockwell et al., 2017; Dixon et al., 2012). Another critical origin is membrane-associated NADPH oxidases which transfer electrons to oxygen. The third pathway involves iron-catalyzed reactions, particularly the Fenton reaction, in which ferrous iron (Fe^2+^) converts hydrogen peroxide into highly reactive hydroxyl radicals. Lipid metabolism also contributes to ROS generation through polyunsaturated fatty acid (**PUFA**) oxidation producing lipid hydroperoxides (Juan et al., 2021). Together, these pathways constitute a dynamic network for ROS production, when unchecked by antioxidant systems, promotes oxidative stress and drives ferroptosis.

**(3) Lipid metabolism:** Lipid metabolism is dynamically regulated by multiple pathways including the synthesis, influx and efflux, storage, and degradation which are essential for cellular membrane structure, energy balance, and signaling (Chandel, 2021). Phospholipids containing PUFAs are main components of cellular membranes determining membrane fluidity and susceptibility to oxidative damage. Inside cells, lipids can be stored as neutral lipids in lipid droplets or mobilized through lipolysis and β-oxidation to meet cellular energy demands. As mentioned above, lipid metabolism is closely linked to ROS production, and dysregulated lipid metabolism can promote lipid peroxidation and contribute to ferroptosis.

**(4) Antioxidant pathways:** Antioxidant system comprises a network of enzymatic and non-enzymatic defenses that maintain ROS homeostasis by neutralizing ROS and prevent oxidative damage (Huchzermeyer et al., 2022). Central to this system is glutathione (**GSH**), acting as a cofactor for glutathione peroxidase 4 (**GPX4**), reducing lipid hydroperoxides to non-toxic lipid alcohols and protects membrane integrity (Weaver and Skouta, 2022). Other key enzymatic antioxidants include superoxide dismutase (**SODs**), catalase, and peroxiredoxins, which convert superoxide and hydrogen peroxide into less reactive molecules. Non-enzymatic antioxidants such as vitamin E, coenzyme Q10 (**CoQ10**), and NADPH also contribute to maintaining ROS balance and regenerating antioxidant capacity (Manful et al., 2025). When these defense systems are compromised, such as during GSH depletion, GPX4 inactivation, or impaired NADPH supply, cells become vulnerable to excessive lipid peroxidation and oxidative stress, ultimately promoting ferroptosis.

## 3 The effects of abused drugs on ferroptosis pathways

Emerging evidence suggests that ferroptosis acts as a bridge linking oxidative stress, neuroinflammation, and neuronal loss in multiple neurodegenerative diseases. Briefly, dysregulated iron homeostasis in the brain elevates the labile iron pool, promoting the Fenton reaction (ROS generation) that oxidizes PUFA-containing phospholipids, leading to irreversible membrane damage. This process is exacerbated by impaired antioxidant defenses. Ferroptosis interacts closely with neuroinflammatory pathways: damaged cells can release danger-associated molecular patterns (**DAMPs**), which activate microglia, increase the production of pro-inflammatory mediators, and further propagate oxidative stress, creating a self-reinforcing cycle of neuronal injury and inflammation. Previous investigations have shown that various types of abused drugs could interact with and affect iron metabolism, ROS production, lipid metabolism, and the antioxidant system. Therefore, it is highly possible that ferroptosis serves as a common mechanism underlying drug-induced neuroinflammation and neuronal injury although the hypothesis is still in embryo stage. Here, we summarize the effects of the six most common abused drugs on ferroptosis-relevant pathways, suggesting that targeting ferroptosis could be a novel therapeutic approach for mitigating neurological complications associated with SUDs.

### 3.1 The effects of alcohol on ferroptosis-related pathways

Alcohol use disorders (**AUDs**) have been a major public health concern worldwide, imposing substantial social and economic burden on modern society. In Western (high-income) countries, the total economic cost of alcohol and its related diseases have been estimated to reach around 1-2% of GDP, including healthcare expenditures, productivity losses, and criminal justice costs. AUDs have high comorbidity with liver diseases, injuries, cardiovascular diseases, cancers, and a variety of mental health disorders, which disproportionately affect young and middle-aged adults. Epidemiological studies have shown that 52.8% of the adult population consumes alcohol regularly (CDC, 2026), and approximately 178,000 people die from alcohol-attributable causes annually in the United States alone (Esser et al., 2024). Alcohol is well known to activate microglia through multiple neuroimmune signaling pathways (Mayfield et al., 2013). Alcohol has been extensively investigated for its close association with ferroptosis, and it can create a strong pro-ferroptosis environment in the brain which is associated with AUDs.

#### 3.1.1 Alcohol effects on iron homeostasis

Alcohol abuse is a risk factor related to brain iron accumulation across adulthood (Gustavsson et al., 2024). Alcohol increases intracellular iron concentration through multiple mechanisms. Alcohol and its metabolite acetaldehyde induce oxidative and inflammatory signaling leading to increased hepcidin expression in microglia (Nemeth et al., 2004). Functioning as a negative regulator of cellular iron release, hepcidin downregulates ferroportin which impairs iron efflux and promotes iron retention in cells (Agoro and Mura, 2016). Meanwhile, alcohol can increase TF expression and promote ferritinophagy, a selective form of autophagy where cells degrade ferritin (Kohgo et al., 2005; Zhao et al., 2021; Xu et al., 2022), which releases stored iron into the labile pool.

#### 3.1.2 Alcohol effects on lipid metabolism

The direct evidence showing the global effects of alcohol on brain lipid profiles is limited. However, alcohol has been shown to exert profound effects on lipid peroxidation through modifying PUFA content in cellular membranes (Pawlosky et al., 2004). Mechanistically, alcohol has been reported to increase the levels of ACSL4 which may enhance PUFA's production and thereby increase susceptibility to lipid peroxidation (Zhao et al., 2021). In parallel, alcohol increases oxidative stress which activates lipoxygenases (**LOXs**) as well as other oxidizing enzymes converting PUFA phospholipids into lipid hydroperoxides (PLOOH). The ability of alcohol to increase lipid peroxidation is evidenced by the elevated levels of several lipoperoxidation markers, including malondialdehyde (**MDA**), 4-hydroxynonenal (**4-HNE**), and C11-BODIPY fluorescence (Zhao et al., 2021; Xu et al., 2022; Mutlu-Turkoglu et al., 2000; Meagher et al., 1999). Interestingly, in PC12 neuronal cells, ethanol dose-dependently enhanced ferrous iron-mediated lipid peroxidation, as measured by cis-parinaric acid oxidation (Sun et al., 1997). Collectively, these findings demonstrate that alcohol robustly promotes lipid peroxidation, creating a biochemical environment favorable for ferroptosis. However, only Xu et al. (2022)) establishes that ferroptotic cell death occurs in alcohol-induced neurotoxicity.

#### 3.1.3 Alcohol effects on ROS production

Alcohol has been widely acknowledged to upregulate oxidative stress and ROS production, mainly through mitochondrial dysfunction, contributing significantly to its pathological effects (Ambade and Mandrekar, 2012; Das, 2007). *In vivo*, ethanol is oxidized by alcohol dehydrogenase generating NADH and increasing the cellular NADH/NAD^+^ ratio. This redox state shift places excessive electron pressure on the mitochondrial ETC, promoting electron leakage and superoxide formation. Superoxide is subsequently converted to H_2_O_2_ generating highly reactive hydroxyl radicals through the Fenton reaction in the presence of ferrous iron (Waddell et al., 2022). In the brain, chronic alcohol enhances ROS production by inducing cytochrome P450 2E1, while acetaldehyde and ROS can cause mitochondrial membranes collapse, further exacerbating electron leakage (Howard et al., 2003). These radicals initiate the peroxidation of PUFAs through ACSL4, leading to the formation of lipid radicals.

#### 3.1.4 Alcohol effects on antioxidant system

Under physiological conditions, lipid peroxides can be detoxified by the GSH-GPX4 antioxidant system. However, alcohol-induced depletion of glutathione has been shown to impair this protective pathway in alcoholic liver disease models, allowing lipid ROS to accumulate and potentially promoting ferroptosis oxidative damage (Luo et al., 2023; Yu et al., 2026). In these same models, ethanol-induced cell death was effectively reversed by the canonical ferroptosis inhibitors, such as Ferrostatin-1 (Luo et al., 2023; Yu et al., 2026). Alcohol can also inhibit the cystine/glutamate antiporter system Xc^−^ (SLC7A11/SLC3A2), reducing cystine uptake thereby limiting GSH synthesis both *in vitro* and *in vivo* (Xu et al., 2022; Yu et al., 2026; Zhu et al., 2025). In the mouse model, alcohol induced neuronal damage in the hippocampus and prefrontal cortex and suppressed major anti-ferroptotic pathway GPX4 which was then reversed with the ferroptosis inhibitor Ferrostatin-1 (Xu et al., 2022). In addition, alcohol affects auxiliary ferroptosis-modulating systems. Nuclear factor erythroid 2-related factor (**NRF2**) is a master regulator of antioxidant and iron-handling genes including SLC7A11, GPX4, ferritin, ferroportin 1, heme oxygenase-1 (**HO-1**). Alcohol may decrease NRF2 which blunts cellular capacity to buffer iron and detoxify ROS/lipid peroxides (Wu et al., 2012). Alcohol can also impair another ferroptosis-suppression system, the ferroptosis suppressor protein 1 (**FSP1**) - CoQ10 axis which sensitizes cells to ferroptosis contributing to fatty liver diseases (Wang et al., 2022). Together with alcohol-induced iron accumulation and enhanced lipid peroxidation, impairment of the antioxidant systems is consistent with activation of ferroptosis-related pathways. Nevertheless, direct evidence demonstrating ferroptotic neuronal death in alcohol-exposed brain tissue remains limited, and further investigation is needed to establish ferroptosis as a causal mechanism underlying alcohol-induced neurotoxicity.

### 3.2 The effects of METH on ferroptosis-related pathways

METH is a potent psychostimulant that produces profound neurotoxic effects, particularly within dopaminergic regions such as the striatum and basal ganglia. Chronic METH abuse is strongly associated with neuroinflammation, progressive dopaminergic degeneration, and an increased risk of neurodegenerative disorders (Jayanthi et al., 2021; Kousik et al., 2014). Although ferroptosis has not yet been definitively established as the primary mechanism underlying METH-induced neurotoxicity, accumulating evidence demonstrates substantial overlap between METH pathology and canonical ferroptotic pathways.

#### 3.2.1 METH effects on iron homeostasis

METH can disrupt cerebral iron homeostasis, particularly in dopaminergic brain regions. Chronic METH can induce iron accumulation in the basal ganglia region of rodent brains and non-human primates (Hu et al., 2023; Melega et al., 2007), as well as in the BV2 microglia cell line (Lin et al., 2023). In rodents, METH-induced iron accumulation and associated ferroptotic changes were attenuated by liproxstatin-1 and the iron chelator deferiprone (Hu et al., 2023), whereas ferrostatin-1 reduced intracellular iron accumulation in BV2 cells (Lin et al., 2023). METH disrupts blood-brain barrier (**BBB**) integrity, increasing permeability through Nox-2 mediated oxidative stress, inflammatory signaling, and tight junction proteins degradation (Ramirez et al., 2009; Northrop et al., 2016). This compromised barrier may facilitate the abnormal entry of circulating iron and iron-containing proteins into the brain parenchyma. METH may also dysregulate iron homeostasis indirectly through hypoxia-inducible factor 1 (**HIF-1**)- linked pathways which reduce ferroportin levels and subsequently decreased iron efflux (Yan et al., 2022). Altogether, these alterations elevate intracellular labile iron which promotes pro-ferroptotic environment.

#### 3.2.2 METH effects on lipid metabolism

METH has also been shown to globally remodel lipidome in rodent brains (Jiang et al., 2017). METH can robustly increase cytosolic dopamine which readily undergoes autoxidation, producing dopamine quinones and ROS that attack PUFAs (Yamamoto and Raudensky, 2008). Several experimental studies have demonstrated that chronic METH exposure induces lipid peroxidation in the brain. Early studies showed increased MDA in the rodent striatum following METH administration, consistent with oxidative damage to neuronal membrane lipids (Acikgoz et al., 1998; Jayanthi et al., 1998). Elevated serum MDA has also been reported in METH-dependent patients, although this marker is neither brain-specific nor indicative of a particular cell death pathway (Najafi et al., 2017). More recently, chronic METH administration has been shown to induce iron accumulation in the nigrostriatal system together with increased levels of the lipid peroxidation product 4-HNE and MDA, accompanied by increased SOD expression (Hu et al., 2023). Notably, in this model, treatment with iron the chelator deferiprone attenuated METH-induced lipid peroxidation and reversed SOD changes, providing stronger evidence that METH-induced lipid peroxidation is linked to iron-dependent oxidative injury.

ADDIN EN.CITE (Hu et al., 2023; Acikgoz et al., 1998; Jayanthi et al., 1998; Najafi et al., 2017).

#### 3.2.3 METH effects on ROS production

METH can significantly increase ROS production through multiple pathways. Firstly, METH dramatically increases cytosolic dopamine which undergoes spontaneous oxidation to generate superoxide, hydrogen peroxide, and dopamine quinones (Yamamoto and Raudensky, 2008). Dopamine oxidation has been shown to cause mitochondrial swelling and opening of the mitochondrial permeability transition pore (**mPTP**) leading to ETC inhibition (Khan et al., 2005). In microglia, METH induces mitochondrial depolarization and cytochrome *c* release, triggering the intrinsic apoptotic pathway (Sharikova et al., 2018). METH-induced cytochrome *c* release is intimately tied to a destructive feedback loop that dramatically increases the production of ROS (Mashayekhi et al., 2014). Although mitochondrial dysfunction has been extensively documented following METH exposure, direct ultrastructural assessment of mitochondrial morphology remains surprisingly limited. One study has systematically characterized METH-induced mitochondrial ultrastructure by transmission electron microscopy (TEM), demonstrating mitochondrial swelling, cristae disruption, and membrane damage in PC12 cells (Lenzi et al., 2022). More recently, electron microscopy of the mouse hippocampus following chronic METH exposure revealed mitochondrial fragmentation, cristae loss, and membrane disruption (An et al., 2026). However, these alterations are only partially consistent with the canonical ferroptotic mitochondrial phenotype, which is characterized by small electron-dense mitochondria. Consequently, although METH-induced mitochondrial dysfunction likely contributes to oxidative stress and may increase susceptibility to ferroptosis, direct evidence demonstrating ferroptosis-specific mitochondrial remodeling following METH exposure remains limited and warrants further investigation.

#### 3.2.4 METH effects on antioxidant systems

METH can also impair cellular antioxidant defenses, thereby increasing susceptibility to oxidative lipid damage.

Studies in human METH users and experimental models have demonstrated disruption of glutathione-dependent antioxidant systems, including reduced GSH availability and altered activity of antioxidant enzymes such as GPX4, catalase, and SOD (Mirecki et al., 2004). In neuronal cells, METH exposure decreased GPX4 expression that was partially reversed by selenium supplementation (Barayuga et al., 2013). Similarly, chronic METH administration in mice reduced GPX4 expression while increasing iron accumulation and lipid peroxidation; treatment with the iron chelator deferiprone restored GPX4 expression, whereas liproxstatin-1 attenuated METH-induced neurodegeneration (Hu et al., 2023). In murine microglial cells, METH-induced ferroptotic death was associated with suppression of the NRF2/SLC7A11 pathway, resulting in impaired cystine uptake and subsequent reduced GSH availability, and thus compromising GPX4 protection. Activation of this pathway or treatment with ferrostatin-1 rescues cells from METH-induced ferroptotic injury (Lin et al., 2023). However, additional studies using ferroptosis-specific lipid ROS assays or manipulation of ferroptosis regulators are needed to fully establish ferroptosis as a predominant mechanism of METH-induced neurotoxicity.

### 3.3 The effects of cocaine on ferroptosis-related pathways

Cocaine is a potent psychostimulant, and chronic cocaine use is strongly associated with neurotoxicity, neuroinflammation, and progressive neuronal dysfunction. Cocaine abusers have increased risk of multiple neurodegenerative disorders, including Alzheimer's disease and Parkinson's disease (Cai et al., 2016), as well as impaired neurodevelopment (Davis et al., 2025). Cocaine can cause several pathological hallmarks including oxidative stress, mitochondrial dysfunction, and chronic neuroinflammation. Although cocaine has not yet been conclusively established as a direct inducer of ferroptosis, its molecular effects substantially overlap with pathways known to promote iron-dependent lipid peroxidation and ferroptotic cell death.

#### 3.3.1 Cocaine effects on iron homeostasis

Cocaine can directly cause iron overload in the brains of addicts (Adisetiyo et al., 2019; Ersche et al., 2017). Mechanism studies showed that cocaine increased BBB permeability by upregulating matrix metalloproteinases (**MMP**)-2 and MMP-9, and inflammatory mediators, together leading to degradation of critical tight junction proteins zonula occludens-1 (**ZO-1**) as well as other junctional adhesion molecules (Sajja et al., 2016). This compromised barrier facilitates the abnormal entry of circulating iron or iron-containing proteins into the brain parenchyma (Dietrich, 2009).

#### 3.3.2 Cocaine effects on lipid metabolism

Cocaine can cause profound alterations in lipid metabolism in the brain. Cocaine decreased the activity of brain phospholipid metabolic enzymes in dopamine-rich areas (Ross and Turenne, 2002; Ross et al., 2002). Cocaine metabolites possess strong oxidation potential and directly stimulate membrane lipid peroxidation (Wen et al., 2022). Recent evidence further demonstrates that cocaine induces substantial lipid droplet accumulation in microglia both *in vitro* and *in vivo*. Cocaine upregulates multiple lipogenic regulators, including perilipin-2, sterol regulatory element-binding protein 1 and 2 (SREBP1/2), 3-hydroxy-3-methylglutaryl-CoA reductase (HMGCR), diacylglycerol O-acyltransferase 1 (DGAT1), thereby enhancing neutral lipid storage and remodeling cellular lipid pools (Cheng et al., 2026). Lipid droplets serve as reservoirs of PUFAs and are highly susceptible to peroxidation. Consequently, cocaine-induced lipid remodeling may enrich microglia with oxidizable phospholipids, sensitizing these cells to ferroptotic lipid damage. Of note, the effects of cocaine on microglial lipid metabolism were still preliminary and had not been repeated in other labs in *in vivo* models beyond microglia.

#### 3.3.3 Cocaine effects on ROS production

Cocaine has been widely recognized as a potent stimulator for ROS production *in vitro* and *in vivo*. It can increase intracellular calcium influx which enhances electron leakage from the ETC, leading to excessive superoxide generation (Cunha-Oliveira et al., 2013). In parallel, cocaine metabolites directly oxidize membrane lipids, further amplifying lipid ROS accumulation (Wen et al., 2022). Cocaine also robustly activates redox-sensitive inflammatory pathways: it stimulates NF-κB signaling, increasing production of pro-inflammatory cytokines, including TNFα, IL1β, and MCP-1, as well as oxidative stress in microglia (Lopez-Pedrajas et al., 2015). Collectively, these mechanisms establish a highly oxidative and inflammatory microenvironment that predisposes neural cells to ferroptotic degeneration.

#### 3.3.4 Cocaine effects on antioxidant systems

Compared to the effects of cocaine on ROS production, the direct evidence revealing cocaine effects on antioxidant system is relatively scarce. However, cocaine can indeed impair cellular antioxidant defenses. In rodent models, cocaine decreases the reduced glutathione-to-oxidized glutathione (GSH/GSSG) ratio, indicating depletion of the major intracellular antioxidant reservoir (Lopez-Pedrajas et al., 2015).

### 3.4 The effects of nicotine on ferroptosis-related pathways

Tobacco use and nicotine exposure have significant harmful effects on human health, impacting nearly every organ in the body. Nicotine is a highly addictive chemical that alters brain chemistry, leading to dependence and making it difficult for users to quit. When tobacco is burned, it releases thousands of chemicals, including tar and carbon monoxide, many of which are toxic and carcinogenic. Tobacco use has been increasingly linked to ferroptosis. In general, components of tobacco smoke, such as ROS, free radicals, and heavy metals, can disrupt cellular redox balance and promote oxidative stress, creating conditions that trigger ferroptosis. However, the effects of nicotine on ferroptosis are complex and context dependent. In certain circumstances, nicotine also showed anti-ferroptosis effects (seen below discussions). The obviously contradictory results might be derived from the parameters of nicotine exposure such as dose (low vs. high; duration of exposure (short vs long-term), cell types) or different mechanisms were involved in (receptor-mediated vs. non-receptor mediated effects).

#### 3.4.1 Nicotine effects on iron homeostasis

The effects of nicotine on brain iron homeostasis are complex and sometimes inconsistent. Previous studies suggested that nicotine can induce brain iron accumulation through dysregulation of iron transport (Kaisar et al., 2018). In addition, nicotine can increase BBB permeability by altering tight junction proteins, potentially facilitating greater entry of circulating iron and other pro-oxidant molecules into the CNS (Hawkins et al., 2004; Hutamekalin et al., 2008). However, other studies indicated that nicotine could also have protective effects against iron-mediated toxicity. For example, nicotine was shown to have radical scavenging properties *in vitro* that may limit Fenton-catalyzed oxidative damage (Malczewska-Jaskola et al., 2016; Ferger et al., 1998). In rotenone-mediated rat model of Parkinson's diseases, long-term intermittent exposure to nicotine protected against the dopaminergic neuron degeneration. Mechanistical study showed that nicotine could activate the nicotinic acetylcholine receptor (nAChR) which leads to the reduction in the labile iron pool (Mouhape et al., 2019). Overall, these findings suggest that nicotine can either exacerbate or mitigate iron-associated neuronal injury depending on the experimental context, exposure paradigm, and disease models.

#### 3.4.2 Nicotine effects on lipid metabolism

Nicotine's effects on lipid metabolism remain also controversial. Acute nicotine exposure was shown to increase oxidative stress markers in rat brain mitochondria. However, chronic nicotine significantly induced mitochondrial glutathione S-transferase A4-4 (GSTA4-4), an enzyme that preferentially detoxifies reactive α, β-unsaturated aldehydes, particularly 4-HNE (Bhagwat et al., 1998). These findings may suggest that acute nicotine promotes mitochondrial lipid peroxidation, while the induction of GSTA4-4 likely represents an adaptive protective response to elevated oxidative stress under chronic conditions. In addition, nicotine has been shown to exert biphasic effects in rat neuronal systems dependent on the doses. In PC12 cells, low concentrations of nicotine reduce oxidative stress and lipid peroxidation, whereas higher doses increase ROS and enhance membrane lipid oxidation which implied that nicotine's effects on lipid homeostasis are dose-dependent (Guan et al., 2003). There is direct linkage between nicotine signaling and membrane phospholipid turnover. In microglia, nicotine influence phospholipid metabolism through receptor-mediated signaling pathways. Activation of α7 nAChRs stimulates phospholipase C, which hydrolyzes membrane phosphatidylinositol 4,5-bisphosphate into the second messenger inositol trisphosphate and diacylglycerol (Suzuki et al., 2006). All these findings suggest that modulation of phospholipid metabolism by nicotine could potentially influence susceptibility to lipid peroxidation and subsequent ferroptotic cell death.

#### 3.4.3 Nicotine effects on ROS production

Similarly, nicotine exerts complex effects on ROS homeostasis in the brain, reflecting both pro-oxidant and antioxidant actions depending on the experimental context. Early work demonstrated that nicotine could induce substantial oxidative stress in isolated brain mitochondria, increasing ROS production (Bhagwat et al., 1998). This pro-oxidant effect is further substantiated by nicotine-mediated upregulation of CYP2E1 (Howard et al., 2003). CYP2E1 is a known ROS-generating enzyme, and its upregulation in smokers and alcoholics suggests a sustained increase in the production of superoxide and hydrogen peroxide. On the other hand, nicotine directly interacts with mitochondrial complex I of ETC and reduces electron leak at this site, thereby decreasing ROS formation (Cormier et al., 2003). These findings were supported by another study showing that nicotine helps maintain the intramitochondrial redox state (Xie et al., 2005). In models of dopaminergic neurotoxicity induced by 6-hydroxydopamine (6-OHDA), oxidative stress is markedly elevated through lipid peroxidation; however, nicotine exhibits only limited neuroprotective capacity in this context (Soto-Otero et al., 2002). Moreover, nicotine does not prevent mitochondrial dysfunction or collapse induced by MPP+ or calcium overload (Xie et al., 2005). Overall, these findings suggest that nicotine does not act as a straightforward pro-oxidant or antioxidant agent but rather as a regulator and the outcomes are likely context dependent.

#### 3.4.4 Nicotine effects on antioxidant system

Nicotine has dual effects on cellular antioxidant defenses. In HIV-1 transgenic rats, nicotine upregulated the expression of genes involved in GSH-related defense and ROS detoxification in a region-specific manner (Song et al., 2016). Lower doses of nicotine reduced oxidative injury, whereas higher concentration produced the opposite effects (Guan et al., 2003). Of note, it remains unclear whether these antioxidant effects were directly induced by nicotine or instead reflected a compensatory response to increased oxidative stress. Nicotine was shown to directly scavenge reactive radicals and protects against oxidative damage in several neurodegeneration rodent models, including 6-OHDA toxicity and experimental paradigms relevant to Parkinson's and Alzheimer's diseases (Soto-Otero et al., 2002; Linert et al., 1999). Mechanistic study showed that NRF2/HO-1 axis through α7 nAChR receptor plays critical roles in nicotine mediated protection against microglial oxidative stress linking nicotine signaling and ferroptosis resistance (Parada et al., 2013).

Collectively, the available evidence suggests that nicotine exhibits biphasic effects on ferroptosis-related pathways. Here we propose a conceptual framework explaining why different studies may obtain different results. At relatively low concentrations or during chronic intermittent exposure, activation of α7 nAChR receptor may stimulate adaptive antioxidant pathways, including NRF2/HO-1 signaling and maintenance of mitochondrial redox homeostasis, thereby limiting lipid peroxidation and ferroptotic susceptibility. Conversely, high-dose or acute nicotine exposure may overwhelm these protective mechanisms through rapid ROS production, CYP2E1 induction, disruption of iron homeostasis, and enhanced lipid peroxidation, ultimately promoting ferroptosis. In addition, cell-type-specific differences in nicotinic receptor expression and intrinsic antioxidant capacity may further contribute to the divergent findings reported across neuronal, glial, and disease models. Future studies directly comparing nicotine dose, exposure duration, receptor dependence, and cell type under standardized ferroptosis assays will be necessary to test these hypotheses and clarify nicotine's role in ferroptotic neurodegeneration.

### 3.5 The effects of cannabis on ferroptosis-related pathways

In recent years, cannabis-derived products have become increasingly available for both medicinal and recreational use. Cannabis sativa contains hundreds of phytocannabinoids, many of which have been investigated for their neuroprotective and neurodegenerative properties (Stone et al., 2020). Among these, cannabidiol (**CBD**) and Δ9-tetrahydrocannabinol (**THC**) are the most extensively studied and have demonstrated antioxidant, anti-inflammatory, and antiapoptotic effects in numerous experimental models of neurodegeneration. However, like nicotine, the effects of cannabinoids on the brain's oxidative environment appear to be highly context-dependent, varying with cannabinoid species, concentration, exposure duration, and disease models. Consequently, their potential relationship with ferroptosis remains incompletely understood.

#### 3.5.1 Cannabis effects on iron homeostasis

Direct evidence linking cannabinoids to brain iron is very limited. Nevertheless, several studies suggest that CBD can modulate intracellular iron concentration, particularly under conditions of pathological iron overload. In rodent models of brain iron accumulation, CBD restores mitochondrial ferritin levels, attenuates mitochondrial dysfunction, and reduces iron-induced neuronal injury (Silva et al., 2018). Mitochondrial ferritin sequesters redox-active iron within mitochondria and reduces the labile iron pool available for Fenton chemistry. These findings suggest that CBD may indirectly oppose ferroptosis by stabilizing mitochondrial iron homeostasis, although direct regulation of ferroptosis-specific iron proteins such as TfR has not yet been established.

#### 3.5.2 Cannabis effects on lipid metabolism

Collective evidence indicates that CBD could inhibit lipid peroxidation in various types of cells or in tissues. CBD significantly reduces β-amyloid-induced lipid peroxidation and improves cell survival in cultured PC12 neuronal cell (Iuvone et al., 2004). CBD also dose-dependently reduces MDA levels in chicken embryonic brain tissue, supporting its ability to protect membrane PUFAs from oxidative damage (Muchacka, 2025). Unlike other abused drugs, CBD has not yet been shown to upregulate key lipid-remodeling enzymes such as ACSL4 or lysophosphatidylcholine acyltransferase 3 (**LPCAT3**).

#### 3.5.3 Cannabis effects on ROS production

Previous investigations have indicated the therapeutic potential of cannabinoid derives from their antioxidant ability and mitochondrial protection across various types of neural cells. In cultured hippocampal neurons, low concentrations of CBD reduce H_2_O_2_-induced ROS production and improve neuronal survival (Kim et al., 2021). Similar antioxidant effects have been observed in astrocytes, where CBD attenuates oxidative stress and preserves cellular viability (di Giacomo et al., 2020). In differentiated neurons, CBD was more effective at mitigating H_2_O_2_-induced oxidative damage and maintaining mitochondrial function, while THC provided less protection in a dose-dependent manner (Raja et al., 2020). CBD can also reduce neuroinflammatory ROS production in microglia by blocking NADPH oxidase and inhibiting downstream NF-κB signaling pathways (Dos-Santos-Pereira et al., 2020). Consistently, CBD reduces iNOS and IL1β expression and decreases NO and pro-inflammatory cytokine release in PC12 cells (Esposito et al., 2006). Additionally, through inhibition of voltage-gated calcium channels, cannabinoids can limit mitochondrial calcium overload leading to less mitochondrial ROS production and subsequent reduced lipid peroxidation (Twitchell et al., 1997). Cannabinoids can also directly modulate ETC activity of complex I, although this depends heavily on experimental conditions such as degree of its inhibition (Singh et al., 2015). On the other hand, one study found that acute and chronic CBD exposure increased mitochondrial complex activity (Valvassori et al., 2013). Overall, available evidence indicates that CBD may function as an anti-ferroptotic agent by maintaining mitochondrial integrity, decreasing ROS production, and preventing lipid peroxidation. However, these findings remain largely circumstantial because most studies have focused on general oxidative stress rather than ferroptosis-specific mechanisms.

#### 3.5.4 Cannabis effects on antioxidant defenses

CBD can robustly enhance the activity of antioxidant systems in various types of *in vitro* and *in vivo* models. In hippocampal neurons subjected to oxygen-glucose deprivation/reoxygenation, CBD prevented GSH depletion and restored GPX activity (Sun et al., 2017). Similarly, in developing chicken embryos, CBD significantly increases GPX and SOD activities in brain tissue mitigating oxidative damage (Muchacka, 2025). The observed enhancement of GSH-dependent antioxidant capacity strongly suggests that CBD reinforces the major anti-ferroptotic defense network. However, none of the studies discussed above directly evaluated canonical ferroptosis markers, such as GPX4, SLC7A11, ACSL4, iron-dependent phospholipid peroxidation, or mitochondrial changes. More recent studies have begun to address this gap by demonstrating that CBD directly suppresses experimentally induced ferroptosis. In neuronal SH-SY5Y cells, CBD activated the NRF2 signaling pathway and preserved GPX4 expression, as well as maintained ferroportin levels (Klimiuk et al., 2026). Outside of nervous system, CBD rescued cell viability and restored GPX4 activity in response to classical ferroptosis inducers, including RSL3 and erastin, in human chondrocytes (Wipplinger et al., 2025). Nevertheless, additional studies in primary neurons, astrocytes, microglia, and *in vivo* models are required to establish whether inhibition of ferroptosis represents a plausible mechanism underlying CBD-mediated neuroprotection.

### 3.6 The effects of opiate-like drugs on ferroptosis-related pathways

Opioid use disorder (**OUD**) is a chronic, relapsing condition which is associated with significant morbidity and mortality, including a high risk of overdose and death, particularly with the increasing prevalence of potent synthetic opioids like fentanyl. Opiate-like drugs, including morphine and related μ-opioid receptor agonists, have been increasingly linked to ferroptosis modulation (Chen et al., 2019; Huang et al., 2024). Mechanistically, opioid exposure can have profound effects on iron metabolism, lipid metabolism, ROS production, and antioxidant systems leading to increased ferroptosis.

#### 3.6.1 Opiate effects on iron homeostasis

Accumulating evidence, mostly from morphine studies, indicates that opioids can disrupt intracellular iron trafficking, thereby creating a cellular environment favorable for ferroptosis.. Morphine activates μ-opioid receptor-dependent iron redistribution from endolysosomal compartments into mitochondria in neuronal cells, resulting in mitochondrial iron accumulation, elevated ROS production, and cell death (Halcrow et al., 2023). Consistently, morphine-induced modulation of endolysosomal iron resulted in increased levels of ferritin heavy chain in cortical neurons, an effect dependent on the endolysosomal iron transporter DMT1 (Nash et al., 2019; Irollo et al., 2023). ADDIN EN.CITE (Irollo et al., 2023)Besides morphine, fentanyl has recently also been shown to severely disrupt BBB in HIV-1 Tat-transgenic mice which potentially contributes to the dysregulation of brain iron homeostasis (Rademeyer et al., 2024). Because excessive intracellular iron promotes Fenton chemistry and lipid peroxidation, these findings suggest that opioid-induced iron redistribution could be an upstream mechanism contributing to ferroptotic vulnerability. Importantly, iron chelation prevented morphine-induced ferritin heavy chain upregulation in cortical neurons (Nash et al., 2019). However, whether iron chelation directly prevents morphine-induced ferroptotic cell death requires further investigation.

#### 3.6.2 Opiate effects on lipid metabolism

The direct evidence showing the effects of opiate drugs on lipid metabolism is limited. One study showed that chronic morphine administration could change rat brain lipidomic profiles (Bodzon-Kulakowska et al., 2017). Morphine administration reduced the fatty acid contents in spinal cord and brain through increasing oxidative stress (Ozmen et al., 2007). Heroin administration significantly increased markers of lipid peroxidation MDA in murine brain tissue (Pan et al., 2005).

#### 3.6.3 Opiate effects on ROS production

Opioid exposure (including morphine and morphine-related compounds) increased oxidative stress and cytotoxicity in both dose- and time-dependent manners in human SH-SY5Y cells (Halcrow et al., 2023; Ma et al., 2015) and rat PC12 cells (Oliveira et al., 2002). Additionally, morphine increased intracellular ROS levels in brain endothelial cells leading to mitochondrial dysfunction (Reymond et al., 2022) which robustly amplifies oxidative stress and in combination with iron imbalance, synergistically drive ferroptotic processes. Heroin administration in mice significantly increased oxidative stress markers and DNA damage in the brain regions including the prefrontal cortex and nucleus accumbens (Pan et al., 2005). Therefore, ROS generation could serve as a major contributor to neuronal DNA injury in reward-related brain regions following opioid exposure (Wang et al., 2023).

#### 3.6.4 Opiate effects on antioxidant system

The strongest evidence connecting opioids to ferroptosis comes from disruption of ferroptosis-related antioxidant defenses. Morphine-induced ferroptosis-like cell death was first demonstrated by Chen et al. (2019)) who showed that liproxstatin-1 attenuated morphine tolerance through inhibition of spinal ferroptosis-like cell death. Although this study did not comprehensively exclude other regulated cell death pathways, the pharmacological rescue by a ferroptosis-specific inhibitor provides functional evidence. The effects of opioids on the antioxidant system have been investigated often together with their effects on oxidative stress. For example, morphine exposure in SH-SY5Y cells disrupts the Nrf2/GSH antioxidant network in a context-dependent manner, especially when autophagy is insufficient, leading to a breakdown of ferroptosis resistance mechanisms with GPX4, rather than the xCT (SLC7A11) antiporter involved in morphine-induced ferroptosis (Huang et al., 2024). Additionally, recent evidence demonstrated that morphine-induced spinal ferroptosis was associated with decreased GPX4 expression, whereas restoration of GPX4 activity reduced ferroptotic injury and improved morphine tolerance (Li et al., 2025). Heroin-treated mice showed reductions in major antioxidant enzymes, including SOD, catalase, and GSH-related antioxidant capacity (Pan et al., 2005). These findings suggest that opioids could negatively regulate cellular antioxidant defenses which ultimately increases the likelihood of ferroptosis.

## 4 The potential therapeutic effects of ferroptosis inhibitors on SUDs and future directions

In this review, we collected evidence showing the profound effects of various types of abused drugs on ferroptosis pathways indicating this process might be the shared mechanisms responsible for abused drugs-mediated neuroinflammation and neuronal injuries. Based on numerous preclinical studies across multiple species, we propose a common pathological cascade induced by drugs of abuse. The initial event is disruption of the BBB, which facilitates peripheral iron influx and promotes cerebral iron overload. This is followed by excessive ROS production and lipid peroxidation, ultimately leading to collapse of the antioxidant defense system, particularly ferroptosis-associated pathways. Under conditions of chronic drug exposure, these three pathological processes, iron dyshomeostasis, oxidative stress, and antioxidant failure, may form a self-perpetuating vicious cycle that progressively amplifies microglial activation and chronic neuroinflammation. The resulting hostile neural microenvironment eventually leads to neuronal injury, synaptic dysfunction, and neurodegeneration, manifested as motor, cognitive, and memory impairments commonly observed in individuals with SUDs. At the same time, each drug exerts distinct effects on specific ferroptosis-related pathways, giving rise to both shared and drug-specific neuropathological alterations (Table 1).

**Table 1:** Summary of ferroptosis-related mechanisms and brain pathological outcomes associated with drugs of abuse.

Substance	Primary Ferroptotic Mechanism	Key Brain Pathological Outcome
Alcohol (Ethanol)	Iron accumulation and GPX4 inactivation leading to ferroptotic death	Neuroinflammation, white matter degeneration, hippocampal neuronal loss, cognitive decline, increased susceptibility to neurodegeneration
Meth	Oxidative stress-driven ferroptosis through GPX4 depletion and lipid peroxidation	Dopaminergic neuron degeneration, striatal damage, cognitive impairment, memory deficits, increased risk of Parkinsonian pathology
Cocaine	Iron dyshomeostasis and oxidative stress promoting ferroptotic vulnerability	Basal ganglia iron accumulation, dopaminergic dysfunction, structural brain remodeling, cognitive deficits, enhanced neurodegenerative risk
Opioids (Morphine, Heroin, Fentanyl)	GPX4/GSH pathway suppression and ferroptotic activation	Neuroinflammation, microglial activation, impaired synaptic plasticity, opioid tolerance, cognitive dysfunction
Nicotine	Disruption of iron homeostasis with increased oxidative stress	Oxidative neuronal injury, cerebrovascular dysfunction, BBB disruption, increased vulnerability to neurodegenerative disorders (context-dependent)
Cannabis/Cannabinoids	Context-dependent modulation of ferroptosis and redox homeostasis	Altered synaptic signaling, cognitive impairment with chronic exposure, possible neuroprotection in some experimental models via antioxidant changes

**Figure 2:**
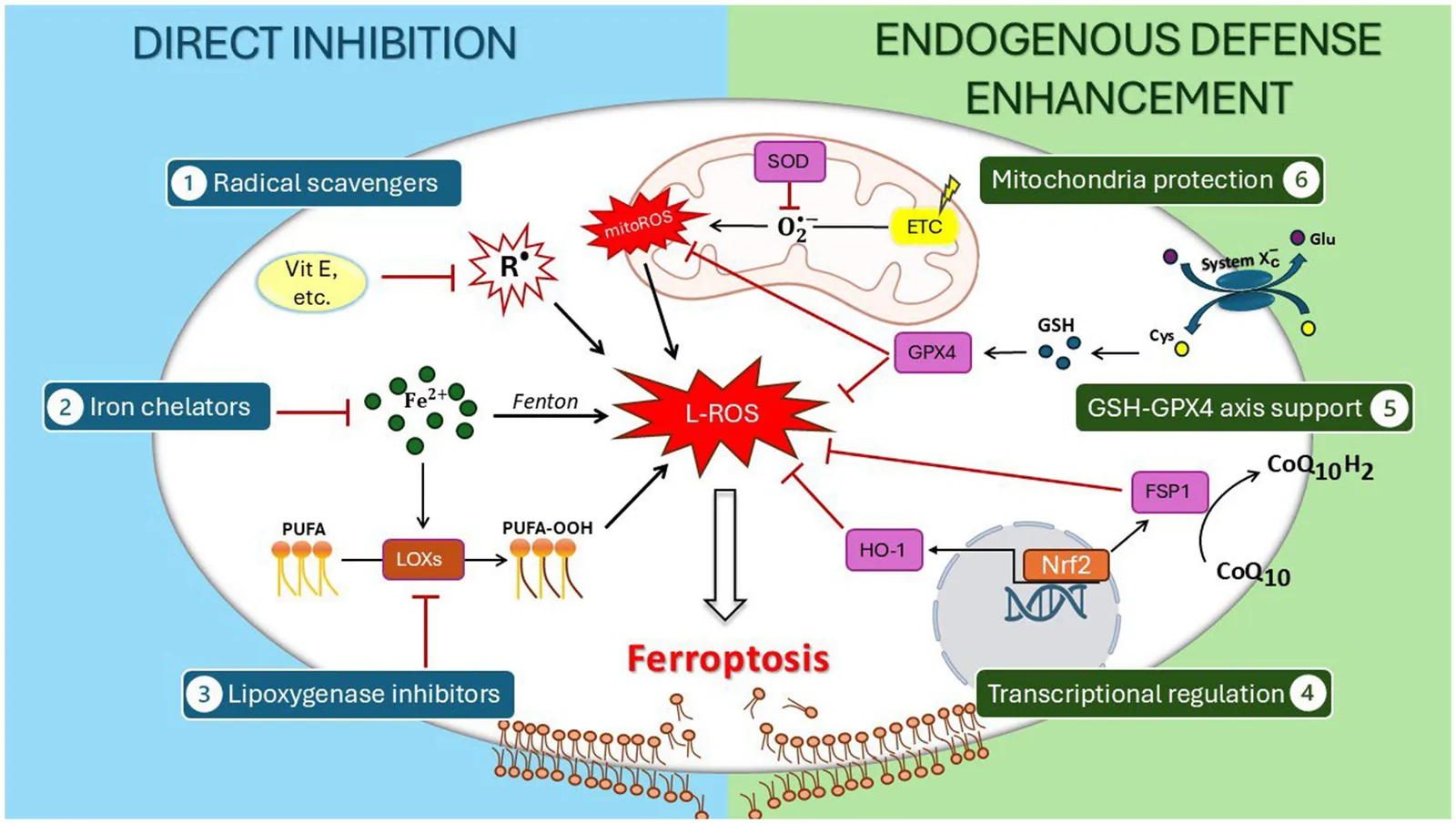
Possible routes of ferroptosis inhibition in SUDs. Anti-ferroptotic interventions can be broadly divided into two categories: (1–3) direct inhibition of lipid peroxide formation and propagation, and (4–6) enhancement of endogenous antioxidant defense systems. (1) Radical scavengers may be broadly beneficial across most drug classes by reducing ROS and lipid radical propagation, although they are less mechanistically specific. (2) Iron chelation may be particularly relevant for AUD, where iron accumulation and dysregulated iron homeostasis are strongly documented. (3) LOX inhibitors may be especially applicable to alcohol-associated ferroptosis or in cocaine-mediated lipidome changes. (4) Transcriptional regulation through NRF2 activation may be particularly important in opioid exposure, where disruption of the NRF2–glutathione antioxidant network has been observed. Impaired FSP1–CoQ10 defense presents a suitable target in AUD. (5) Support of the GSH–GPX4 axis represents a central anti-ferroptotic strategy and may be especially relevant in alcohol-, METH-, and opioid-associated neurotoxicity. (6) Mitochondrial protection may be particularly effective in METH-associated neurotoxicity, which is strongly linked to dopamine auto-oxidation, mitochondrial dysfunction, and amplification of mitochondrial reactive oxygen species (mitoROS).

Therefore, therapeutic strategies aimed at interrupting this feed-forward pathological loop may provide a promising approach for alleviating the neurological abnormalities associated with chronic drug abuse. Indeed, pharmacological inhibition of ferroptosis has shown neuroprotective effects across multiple experimental models of SUDs (Summarized in Table 2). In addition, antioxidant compounds such as N-acetylcysteine, have been extensively reported to attenuate drug-induced neuronal injury, reduce oxidative damage, and preserve dopaminergic function (Smaga et al., 2021; Womersley et al., 2019). Of note, such treatments are mainly beneficial for neurotoxic damage and behavioral rigidity caused by long-term exposure with abused drugs. Whether targeting ferroptosis pathway could reduce the neuropsychological syndromes at the early phase of addiction cycle such as psychoactive effects and craving remain much unexplored until now. Also, current evidence remains largely restricted to *in vitro* and animal studies, and the clinical relevance of ferroptosis in human SUD pathology has yet to be firmly established. Future research should prioritize the development of brain-penetrant, selective ferroptosis inhibitors or inducers of endogenous defense systems (Figure 2), and evaluate their efficacy in reducing neurotoxicity, improving cognitive outcomes, and preventing relapse. Moreover, elucidating the interaction between ferroptosis and other forms of regulated cell death, as well as its role in withdrawal and relapse-related neuroadaptations, will be critical for advancing ferroptosis-targeted strategies as a novel adjunctive approach in the treatment of SUDs.

**Table 2:** Preclinical evidence linking ferroptosis inhibitors to abused drugs.

Drug Class	Ferroptosis Inhibitor(s)	Primary Mechanism of Action	Supporting Preclinical Findings & Targets
Psychostimulants *(Methamphetamine, Cocaine)*	Liproxstatin-1 (Lip-1) (Hu et al., 2023) Ferrostatin-1 (Fer-1)(Hu et al., 2023; Lin et al., 2023) Deferiprone (DFP)(Hu et al., 2023)	Radical-trapping antioxidants (RTAs) iron chelation	•Attenuated neurodegeneration and oxidative neuronal injury •Restored NRF2–SLC7A11–GPX4 pathway •Reduced lipid peroxidation •Reduced intracellular Fe^2+^
Alcohol *(Ethanol)*	Ferrostatin-1 (Fer-1)(Xu et al., 2022; Yu et al., 2026)	RTA	•Attenuated neuronal injury, •Restored GPX4 •Preserved synaptic proteins •Reduced lipid peroxidation
Opioids *(Morphine)*	Liproxstatin-1 (Lip-1)(Chen et al., 2019) Deferoxamine (DFO)(Huang et al., 2024) Cordycepin (Li et al., 2025) Naloxone (Huang et al., 2024)	RTA Iron chelation SIRT1 activation μ-opioid receptor antagonist	•Reduced lipid peroxidation •Restored antioxidant defenses •Reduces iron accumulation •Suppressed inflammatory cytokines
